# Behavioral determinants for COVID-19 vaccine acceptance among students, faculty, and staff at a rural public university

**DOI:** 10.1080/21642850.2022.2074007

**Published:** 2022-05-13

**Authors:** Sarah Bauler, Adam Hege, Tom Davis, Emilee Schluth, Caroline Pruitt, Victoria Moreno, Monica Verhaeghe, Erin D. Bouldin

**Affiliations:** a Department of Health and Exercise Science, Appalachian State University, Boone, NC, USA; b World Vision International, Geneva, Switzerland; c Bloomberg School of Public Health, Johns Hopkins University, Baltimore, MD, USA

**Keywords:** COVID-19, vaccine hesitancy, barrier analysis, behavior, determinants, universities

## Abstract

**Background:**

Vaccine hesitancy for COVID-19 is a major obstacle to achieving high vaccine coverage. Low vaccine confidence among college students is one factor fueling the COVID-19 pandemic in the U.S.

**Objective:**

The purpose of this study was to evaluate COVID-19 vaccine hesitancy and barriers to vaccine uptake among students, faculty, and staff at a rural public university.

**Method:**

We used the Barrier Analysis (BA) mixed-methods approach, which explores determinants of the desired behavior using the Health Belief Model and Theory of Reasoned Action. We developed a BA questionnaire and distributed it through Qualtrics to 4,600 randomly selected students (*n* = 4,000), faculty (*n* = 300), and staff (*n* = 300) from March 11 to April 1, 2021. We defined Acceptors as those who were willing to be vaccinated and Non-acceptors as those who were not.

**Results:**

Our analysis found that among Non-acceptors, perceived social norms, perceived negative consequences, and trust had the highest association with COVID-19 vaccine hesitancy among students, faculty, and staff.

**Conclusion:**

These findings illustrate the need to develop effective behavior change strategies for COVID-19 vaccines uptake that identify sources of trusted information among vaccine-hesitant college students, faculty, and staff, while leveraging enablers to increase COVID-19 vaccination coverage on university campuses.

## Introduction

More than 43 million confirmed COVID-19 cases have been reported in the U.S. since the start of the global pandemic, which has led to more than 700,000 deaths (WHO, 2021). The U.S. has about 18% of the world’s cases and 14% of the world’s deaths – the most of any country. Due largely to the COVID-19 pandemic, the U.S. had a decline in life expectancy of 1.5 years, from 78.8 to 77.3 years, from 2019 to 2020 (Arias, Betzaida, Ahmad, & Kochanek, 2021). While several public health strategies (e.g. mask-wearing, social/physical distancing) have been used to mitigate the ongoing pandemic, a COVID-19 vaccine is the only clinical measure to prevent transmission of the SARS-CoV-2 virus and stem the tide of COVID-19 hospitalizations and deaths. Early in 2021, the U.S. introduced three rigorously tested vaccines under FDA emergency use authorization (CDC, 2021a). At the time of writing this paper, over 383 million COVID-19 vaccines have been administered, yet only 54.2% of eligible people age 12 years and older have been fully vaccinated (CDC, 2021b).

Vaccine hesitancy for COVID-19 is a major obstacle to reaching the expected threshold for herd immunity across the nation, with estimates in hesitancy ranging from 2.7% to 26.7% among the unvaccinated as of September 8, 2021 (CDC, 2021b). A lagging vaccination campaign has allowed new COVID-19 variants, such as the highly contagious Delta variant, to surge among the unvaccinated and cause breakthrough infections among the fully vaccinated (Wernau, 2021). However, breakthrough infections rarely cause severe disease, and over 97% of cases due to COVID-19 are among the unvaccinated (Aubrey, 2021). Reluctance, or refusal, to vaccinate may prolong the pandemic and negatively impact global health and the economy (Kohli, Maschio, Becker, & Weinstein, 2021). Notably, the World Health Organization identified vaccine hesitancy as one of the top ten global health threats in 2019 (WHO, 2019).

Low vaccine confidence among individuals aged 20 to 49 years, which includes college students, are the primary group fueling the resurgence of COVID-19 outbreaks within the U.S. and a barrier to achieving desired levels of vaccine coverage and individuals 20 to 49 years, which includes college students, (Monod et al., 2021). An early study at a northeastern U.S. university reported that nearly half of students would not or were not sure if they would be vaccinated (Synnott, 2021), while another study in the northwest U.S. found that more than 90% of students intended to get a COVID-19 vaccine (Graupensperger et al., 2021). A study among students at a southern U.S. university found that 47.5% were vaccine-hesitant (Sharma, Davis, & Wilkerson, 2021). Vaccinations among college and university students, faculty, and staff can serve as a vital component of improving outcomes in a local community, as COVID-19 has led to severe consequences in counties featuring universities and colleges (Leidner, 2021).

As the COVID-19 pandemic impacts the health and well-being of the university faculty, staff, students, and the local community, it is vital to examine behavioral intentions toward vaccination among campus communities. Polls show that vaccine resistance is highest among rural, primarily Republican-led, Christian, and white communities (Monod et al., 2021). White evangelicals have also been found to be more likely to follow debunked claims and conspiracy theories around vaccines (Economist, 2021). The work described here took place at a mid-sized public institution (student enrollment ∼20,000) in a rural county in North Carolina; 94.8% of the population is white and the largest religious tradition is Evangelical Protestant (ARDA, 2010; US Census Bureau, 2021). Thus, the community in which the university is situated reflects many of the characteristics of locations encountering the highest levels of vaccine hesitancy.This study aimed to identify social and behavioral determinants of vaccine hesitancy among students, faculty, and staff and collect information that would inform social and behavior change interventions on campus and the local public health’s strategy to increase vaccine confidence and uptake.

## Method

### Study design

To better understand the determinants of COVID-19 vaccine acceptance at the university, a team of public health faculty and students with relevant expertise developed a Barrier Analysis (BA) questionnaire. The Barrier Analysis approach explores determinants associated with the Health Belief Model (HBM), the Theory of Reasoned Action (TRA), and other factors to identify the most important behavioral determinants of the desired behavior under study – in this case, receiving a COVID-19 vaccine. A key feature of BA is to compare responses between ‘Doers’ or ‘Acceptors’ of the behavior and ‘Non-doers’ or ‘Non-acceptors’ of the behavior.

The BA methodology has been used in 59 low-to-middle-income countries through 38 organizations (including UNICEF) to determine behavioral determinants associated with desired childcare practices (e.g. exclusive breastfeeding, child vaccination) (Davis, et al., 2022). This approach was recently used in Bangladesh, India, Myanmar, Kenya, the Democratic Republic of the Congo, and Tanzania to examine 12 potential behavioral determinants of vaccine COVID-19 acceptance and found statistically significant differences in behavioral determinants of COVID-19 vaccine confidence among the different countries (Kebede et al., 2021). Another recently published study used BA to identify determinants of vaccine hesitancy in Dhaka (Kalam et. al., 2021). The current study team adapted the BA COVID-19 vaccine questionnaire used in lower-middle-income countries (LMICs) to fit the present context, as high-income countries can learn from the experience of LMICs in fighting endemic and pandemic infectious diseases. See Table 1 for a description of key terms and behavioral determinants explored in this study.

**Table 1.** Description of key terms and behavioral determinants explored in this study.

Key terms	Definition
Study behavior	Intended acceptance of COVID-19 vaccines
Target group	University students, faculty, and staff
Details of the behavior	University students, faculty, and staff have the stated intention to get a COVID-19 vaccine when one is available to them free of charge.
Acceptors (Doers)	Respondents who said they were very likely or somewhat likely to get a COVID vaccine if one was available within the next month at the time of the survey.
Non-acceptors (Non-doers)	Respondents who said they were very unlikely, somewhat unlikely, or not sure if they would get a COVID vaccine if one was available within the next month at the time of the survey.
Behavioral determinants explored	
Perceived social norms	The perception that most people important to an individual think that he/she should get a COVID-19 vaccine when it is available. Questions on who approves and disapproves of the respondent getting a COVID-19 vaccine were also assessed under this determinant. Respondents were also asked specifically about the approval by medical staff (doctors and nurses) and community and religious leaders.
Perceived negative consequences	The negative things a person thinks will happen as a result of getting a COVID-19 vaccine (including the perceived disadvantages of getting a COVID-19 vaccine).
Access	The availability of the needed information, products, or services required to do the behavior
Perceived severity	The extent to which a person believes it would be serious if they (or members of their household or dorm) got the disease or problem that the behavior is meant to prevent (e.g. COVID-19 disease in this study).
Perceived action efficacy	The extent to which a person believes that doing the behavior will prevent the disease/problem that the behavior is intended to prevent (in this study, whether one believes that getting a COVID-19 vaccine will prevent future COVID-19 infection).
Perceived divine will	The extent to which a person believes that God approves or disapproves of him/her doing the behavior (e.g. getting a COVID-19 vaccine). In this study, we explored whether people believed that getting COVID-19 was purely a matter of God’s will or chance, or something that they could control.
Culture and Values	The extent to which the respondent can cite cultural rules, values, or taboos that affect whether they do the behavior or not (e.g. get vaccinated with a COVID-19 vaccine in this study).
Trust	The extent to which the respondent trusts private and public sources of information regarding the safety and efficacy of COVID-19 vaccines (e.g. CDC, universities, doctors, nurses).

### Location of the study

This study was conducted at a rural university located in a southern U.S. state. The poverty rate in the county where the university is based (25.3%) (Data USA, 2019) is well over the national poverty rate (9.2%) (Giannarelli, Wheaton, & Shantz, 2021). Students have a much higher level of food insecurity (46.2%) (McArthur, Ball, Danek, & Holbert, 2018) than the general population (14%) of the state where the university is located. University students represent almost one-third (32%) of the county’s population where the university is based. Nearly 95% of the county population is white and the largest religious tradition is Evangelical Protestant (ARDA, 2010; US Census Bureau, 2021). At the time of data collection, 24.3% of the county population 18 years and older had been vaccinated with at least one does of the COVID-19 vaccine (Citizen Times, 2021).

### Development of the study hypotheses

Research has found that low trust in government and public officials is associated with COVID-19 vaccine hesitancy (Wiltse, 2021). Believing that one’s life is in the control of God and health is dependent on chance or fate is associated with reduced motivation to get vaccinated (Upenieks, Ford-Robertson, & Robertson, 2021). Trusting the safety of COVID-19 vaccines and perceiving COVID as a severe disease have also been found to be strong predictors of intentions to be vaccinated (Karlsson et al., 2021). Taking into consideration these research findings, the HBM and TRA theoretical frameworks, along with our study location, we developed the following hypotheses:

*H1: Low trust in university leadership would positively predict vaccine hesitancy*.

*H2: Higher levels of perceived divine will (religious fatalism) would positively predict vaccine hesitancy*.

*H3: Lower levels of perceived consequences of COVID-19 would positively predict vaccine hesitancy*.

### Data collection

The study’s COVID-19 vaccine BA questionnaire was disseminated through Qualtrics on March 11, 2021, and was open through April 1, 2021, to 4,600 randomly selected students (*n* = 4,000), faculty (*n* = 300), and staff (*n* = 300). Respondents were asked whether or not they had received at least one dose of a COVID-19 vaccine. Those respondents who had not yet received at least one dose were asked questions on the perceived severity of COVID-19, perceived susceptibility of getting COVID-19, trust in COVID-19 vaccines, and perceived negative and positive consequences of receiving a COVID-19 vaccine. Open-ended questions were included to better understand enablers and barriers towards vaccine uptake, as well. Respondents (who had not yet received any COVID-19 vaccine) were asked how likely they were to get the vaccine when it was available to them. Those who said they were ‘somewhat likely’ or ‘very likely’ were classified as ‘acceptors’. Those who said they were ‘very unlikely’, ‘somewhat unlikely’, or ‘not sure’ to be vaccinated were classified as ‘non-acceptors’.

In total, 1,753 people completed the survey, including 1,364 students, 197 faculty, and 186 staff (six did not report their affiliation), representing a 38% response rate overall. About one-third of respondents had received at least one dose of a COVID-19 vaccine; this varied substantially by group, with 85% of faculty, 61% of staff, and 23% of students reporting at least one dose. There were 1,120 respondents who had not yet received at least one dose of the COVID-19 vaccine and were included in the study: 1,050 students (77%), 30 faculty (15%), and 73 staff (39%). The university’s Institutional Review Board reviewed the project and determined that it did not meet the definition of research under 45 CFR 26.102(I) because it was part of ‘public health surveillance activities … during the course of an event or crisis that threatens public health’. At the time of the study, COVID-19 vaccines were only available to people aged 65 years and older, people aged 16–64 years with high-risk medical conditions, and essential workers (including healthcare providers and employees at educational institutions, among others).

### Statistical analysis

Data was cleaned and analyzed using a Barrier Analysis data tabulation sheet developed by staff from two international organizations that use BA extensively (World Vision and Food for the Hungry). This tool calculates the Odds Ratio (OR), Estimated Relative Risk (ERR), 95% confidence interval (CI), and the *p*-value related to the differences between Acceptors and Non-acceptors. The tabulation sheet was created in M.S. Excel and uses Chi-square tests. We used an alpha value of *p* < 0.01 to indicate statistical significance. Open-ended responses were grouped together using artificial intelligence (a function of the BA tabulation table) and further classified thematically. We have reported the ERR results to show levels of association with the behavior. The ERR was calculated for Acceptors and Non-acceptors, but we did not disaggregate the data for university staff, employees, and students.

## Results

**Figure 1.**
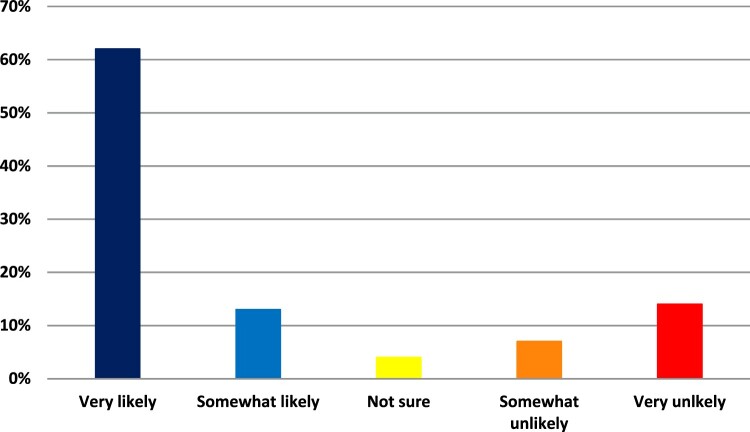
Likelihood of receiving a COVID-19 vaccine in the next month if it was available, as reported by people who had not yet received at least one dose of the vaccine.

Within the study sample (those not yet receiving at least one dose of a COVID-19 vaccine), 840 (75.0%) respondents were classified as Acceptors, and 280 (25.0%) were classified as Non-acceptors. See Figure 1.

### Perceived social norms

Perceived social norms reflect people’s perceptions of what behaviors people typically do or are approved or disapproved by others. Beliefs around both injunctive and descriptive social norms are some of the strongest predictors of vaccine confidence. In this study, Non-acceptors were 79.7 times more likely to say they are not likely to get a COVID-19 vaccine if a doctor or nurse recommends it to them than Acceptors (ERR = 0.01). Surprisingly, Non-acceptors were 19.2 times more likely to say that they have a medical professional in their life who they believe would generally disapprove of them getting a COVID-19 vaccine than Acceptors (ERR = 0.05). Non-Acceptors were 4.2 times more likely to say that most of their close family and friends would not want them to get a COVID-19 vaccine if it was available in the coming month free of charge (ERR = 0.24). When respondents were asked if they have a significant other in their life that they believed would approve or disapprove of their getting a COVID-19 vaccine, Non-acceptors were 5.6 times more likely to say that they had a significant other who would disapprove of them getting a COVID-19 vaccine than Acceptors (ERR = 0.18). Non-Acceptors were also 10.6 times more likely to say they have a professor and 4 times more likely to say they have an employer who would generally disapprove of them getting a COVID-19 vaccine than Acceptors (ERR = 0.09 and ERR = 0.25, respectively).

### Perceived negative consequences

Perceived negative consequences refer to negative things a person thinks will happen as a result of getting a COVID-19 vaccine and includes perceived disadvantages of getting a COVID-19 vaccine. We found that Acceptors were 1.2 times more likely to say that COVID-19 vaccines would be mostly safe than Non-acceptors (ERR = 1.24). Conversely, Non-acceptors were 38.4 times more likely to say that COVID-19 vaccines would ‘not be safe at all’ than Acceptors (ERR = 0.03).

### Access to COVID-19 vaccine information

Preferences for how to access COVID-19 vaccine information differed significantly between Acceptors and Non-Acceptors. Non-acceptors were 1.5 times more likely to say that they prefer to get up-to-date information on COVID-19 through posters and flyers than Acceptors. Non-acceptors were 5.3 times more likely to say that they do not really care to keep up with COVID-19 information than Acceptors.

### Perceived action efficacy

Perceived action efficacy refers to the extent to which a person believes that doing a behavior will prevent the disease/problem that the behavior is intended to prevent. This study asked respondents how confident they were that a COVID-19 vaccine would protect them from contracting the virus. Non-acceptors were 14.4 times more likely to say that they would not be confident at all that a COVID-19 vaccine would protect them from COVID-19 infection than Acceptors (ERR = 0.07).

### Perceived divine will

Perceived divine will refers to the extent to which a person believes that a deity (e.g. God, Allah, the gods) approves or disapproves of him/her doing the behavior, in this case, receiving a COVID-19 vaccine. In this study, we explored whether people believed that getting COVID-19 was purely a matter of God’s will or chance or something that they could control. We found that Acceptors were 1.4. times more likely to strongly agree with the statement, ‘Whether I get COVID-19 is a matter of fate or inevitable. The actions I take will have little bearing on whether or not I get COVID-19’ (ERR = 1.43).

### Culture and values

The culture and values questions explored the extent to which the respondent can cite cultural rules, values, or taboos that affect whether they do the behavior or not. We found that Non-acceptors were 3.5 times more likely to say that there are cultural and religious reasons that they would not get the vaccine than Acceptors (ERR = 0.095). We also found that Acceptors were 2.2 times more likely (than Non-acceptors) to say that getting vaccinated against COVID-19 is a responsibility to protect the health of all (ERR = 2.21), while Non-acceptors were 2.2 times more likely to say that getting vaccinated against COVID-19 is a personal choice (ERR = 0.45).

### Trust in governmental entities promoting COVID-19 vaccines

**Figure 2.**
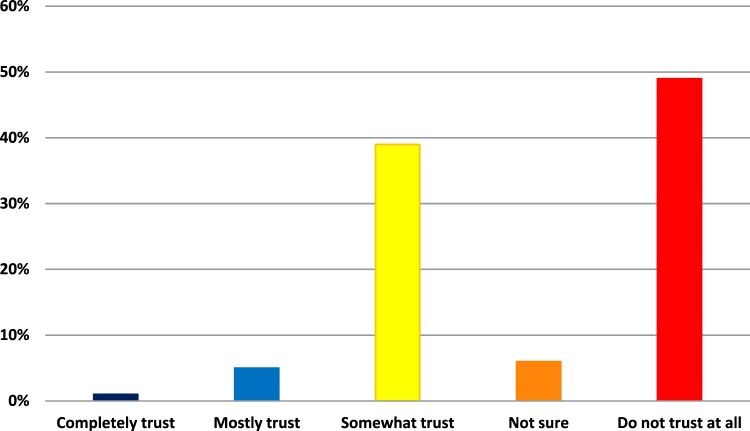
Percentage of Non-acceptors who said they completely trusted (1%), mostly trusted (5%), somewhat trusted (39%), did not trust at all (49%), and were not sure if they trusted (6%) the CDC.

As expected, trust in COVID-19 vaccines differed significantly between Acceptors and Non-Acceptors. Non-acceptors were 31.8 times to say they would not trust COVID-19 vaccines at all than Acceptors (ERR = 0.03). Trust in the information provided on the safety and effectiveness of COVID-19 vaccines from the U.S. Centers for Disease Control and Prevention also differed significantly. Non-acceptors were 6.5 times more likely than Acceptors to say that they ‘do not trust at all’ the information provided by the CDC on the safety and effectiveness of COVID-19 vaccines (ERR = 0.15). Of those respondents who said they are very likely to get a COVID-19 vaccine, 46.5% completely trust the information the CDC provides on vaccine safety and effectiveness. For those respondents who said that they are very unlikely to get a COVID-19 vaccine, less than 1% completely trust the CDC, and 48.9% do not trust the CDC at all. See Figure 2.

**Figure 3.**
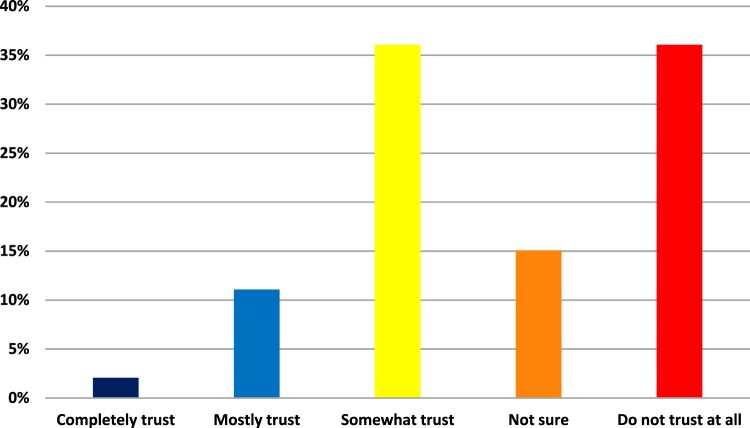
Percentage of Non-acceptors who said they completely trusted (2%), mostly trusted (11%), somewhat trusted (36%), did not trust at all (36%), and were not sure if they trusted (15%) the public university.

We found similar significant differences in levels of trust in the information and guidance provided by the university on the safety and effectiveness of COVID-19 vaccines. Non-acceptors were 5 times more likely than Acceptors to say that they do not trust at all the information provided by the university on the safety and effectiveness of COVID-19 vaccines (ERR 0.20). For those survey respondents who said they were very unlikely to get a COVID-19 vaccine, over 46% of respondents said they do not trust the university at all, and only 2% completely trust the university. For those respondents who were very likely to get the vaccine, 34.8% completely and 49.8% mostly trust the university, and less than 1% did not trust the university at all, as illustrated in Figure 3.

## Discussion

Our study revealed high levels of distrust among Non-acceptors for information provided by the CDC and the university on the safety and effectiveness of COVID-19 vaccines. Students, faculty, and staff who were vaccine hesitant were 31.8 times more likely to say that they do not trust the COVID-19 vaccine information provided by the CDC than respondents who had high confidence in the vaccine. Similarly, Non-acceptors were 5 times more likely than Acceptors to say that they do not trust at all the information provided by the university on the safety and effectiveness of COVID-19 vaccines, although trust in the university was higher than the CDC. For decades, doctors and public health officials have addressed vaccine hesitancy by disseminating CDC vaccine information through various communication channels, which is an approach many universities have used. However, recent research indicates that people who are opposed to vaccinations are more supportive of individual rights and less responsive to people in positions of governmental authority. The research found that skeptics were much more likely to have a highly developed sensitivity for liberty, the rights of individuals, and to have less deference to positions of power than non-skeptics (Tavernise, 2021) Given this, traditional, value-based vaccine-focused messages comprised of facts and information may not be the most effective approach to increase vaccine confidence. These approaches often do not sufficiently address people’s gut feelings towards purity, power, liberty, and trust (Amin et al., 2017).

Perceived social norms were also found to be an important determinant of the respondents’ decision to get a COVID-19 vaccine. In particular, Non-acceptors were less influenced by the recommendation of a doctor or nurse than Acceptors. While healthcare providers continue to be a valued source of COVID-19 information for many people, peer-to-peer vaccine education and promotion may be more effective in increasing COVID-19 vaccine uptake among Non-acceptors in this population. Utilizing peer-to-peer education to increase awareness and uptake of the human papillomavirus vaccine (HPV) was found to be very effective among adolescents (Esaggoff et al., 2019). Hence, identifying trusted and diverse students who can serve as peer mentors and peer promoters, along with personal narratives in favor of COVID-19 vaccines, may achieve similar results in increasing COVID-19 vaccine confidence and uptake among university students, faculty, and staff.

Several other determinants were found to be associated with vaccine acceptance in this population and should be given attention during messaging and vaccine-promotion activities. Those include perceived negative consequences, perceived action efficacy, access to COVID-19 information, perceived divine will, and culture and values. These areas need to be considered and addressed when planning interventions with vaccine-hesitant populations.

Our study findings also indicate that vaccine hesitancy remains a significant barrier to achieving high vaccination levels on a college campus. At the time of the survey (March 2021), 75% of unvaccinated respondents said that they were very likely or somewhat likely to receive a COVID-19 vaccine. However, as of August 31, only 49% of the university students and 87% of faculty and staff have been fully vaccinated (University, 2021). These statistics are alarming for university officials and community leaders, as they indicate that opposition towards COVID-19 vaccines may have gotten stronger over the past few months, despite public health efforts to build vaccine confidence. Moreover, according to the local healthcare system, the COVID-19 vaccination efforts (community clinics, etc.) have stalled as officials grapple with how best to persuade hesitant community members, including college students, to vaccinate (S. Pinnex, personal communication, July 2021).

Low COVID-19 vaccine coverage has led to a surge in cases fueled by the Delta virus throughout campuses in the U.S., which has mobilized some universities to mandate vaccinations to mitigate the spread (Nietzel, 2021). When tailored social and behavior change strategies do not achieve desired COVID-19 vaccine coverage, universities may consider vaccine mandates to increase safety on campus and prevent outbreaks. Under the Supreme Court’s jurisprudence, universities are constitutionally permitted to impose reasonable vaccine mandates (Reiss & DiPaolo, 2021). However, COVID-19 vaccines mandates may prompt backlash, thus mandates should only be considered if contextualized social and behavior change strategies do not adequately contain coronavirus cases on campus (Largent et al., 2020).

### Limitations

Although this study has many valuable and timely implications, the findings presented are from a sample of students, faculty, and staff in N.C. They may not necessarily reflect attitudes and perceptions of COVID-19 vaccines across the nation. However, this study does provide a proof-of-concept for using Barrier Analysis as a tool that can be replicated among college campuses to determine significant barriers and enablers of COVID-19 vaccine uptake among their target population groups. We contend that using formative research tools – like the Barrier Analysis tool – can greatly improve the identification of important determinants of vaccination and other behaviors. This, in turn, can lead to improved effectiveness of social and behavior change strategies designed to improve vaccine uptake and better choices regarding the resources needed for implementing these programs. Finally, a limitation of our study is that we were not able to disaggregate the data for university staff, faculty, and students due to resource constraints for this additional study design and analysis. Exploring differences between behavioral determinants of vaccine confidence could be valuable, as we hypothesize that faculty who were highly vaccine hesitant negatively influenced students’ vaccine intentions.

## Conclusion

To mitigate spikes in COVID-19 cases on campus and bring students fully back into the classroom successfully, universities must build COVID-19 vaccine confidence and facilitate accessible vaccination opportunities among students, faculty, and staff. Conducting formative research using Barrier Analysis and other tools can help assure behavior change campaigns are directed at the most important determinants of vaccine acceptance. Where vaccinations have lagged, there is an urgency to increase COVID-19 vaccine uptake to mitigate the Delta-driven surge of coronaviruses cases and prevent new variants from emerging. Increasing social acceptance of COVID-19 vaccines requires contextualized and tailored behavior change messages and interventions. If these efforts do not reach a sufficient level of COVID-19 vaccine coverage, universities should consider vaccine mandates.

## Data Availability

The data that support the findings of this study are openly available at FAIRshare at https://fairsharing.org/3910.
